# *Wnt11b* Is Involved in Cilia-Mediated Symmetry Breakage during *Xenopus* Left-Right Development

**DOI:** 10.1371/journal.pone.0073646

**Published:** 2013-09-13

**Authors:** Peter Walentek, Isabelle Schneider, Axel Schweickert, Martin Blum

**Affiliations:** Institute of Zoology, University of Hohenheim, Stuttgart, Baden-Württemberg, Germany; University of Colorado, Boulder, United States of America

## Abstract

Breakage of bilateral symmetry in amphibian embryos depends on the development of a ciliated epithelium at the gastrocoel roof during early neurulation. Motile cilia at the gastrocoel roof plate (GRP) give rise to leftward flow of extracellular fluids. Flow is required for asymmetric gene expression and organ morphogenesis. Wnt signaling has previously been involved in two steps, Wnt/ß-catenin mediated induction of *Foxj1*, a regulator of motile cilia, and Wnt/planar cell polarity (PCP) dependent cilia polarization to the posterior pole of cells. We have studied *Wnt11b* in the context of laterality determination, as this ligand was reported to activate canonical and non-canonical Wnt signaling. *Wnt11b* was found to be expressed in the so-called superficial mesoderm (SM), from which the GRP derives. Surprisingly, *Foxj1* was only marginally affected in loss-of-function experiments, indicating that another ligand acts in this early step of laterality specification. *Wnt11b* was required, however, for polarization of GRP cilia and GRP morphogenesis, in line with the known function of Wnt/PCP in cilia-driven leftward flow. In addition *Xnr1* and *Coco* expression in the lateral-most GRP cells, which sense flow and generate the first asymmetric signal, was attenuated in morphants, involving Wnt signaling in yet another process related to symmetry breakage in *Xenopus*.

## Introduction

In vertebrates many inner organs of the chest and abdomen, such as heart and stomach, are asymmetrically localized along the left-right (LR) body axis [1]. Initiation of LR asymmetry in fish, amphibians and mammals is achieved by a cilia-driven leftward flow of extracellular fluids during neurulation [2]–[4]. Ciliated epithelia exist only very transiently and are represented by the amphibian GRP [5], Kupffer’s vesicle in fish [6] and posterior notochord (“node”) in mammals [7], [8]. The lateral margins of these epithelia are characterized by cells which co-express the growth factor *nodal* (*Xnr1* in *Xenopus*) as well as a nodal inhibitor (*Coco* in *Xenopus*) [9]–[12]. As a result of asymmetric flow, the expression of the nodal inhibitor on the left side is down-regulated, which is thought to release nodal repression and initiate the nodal-cascade exclusively in the left lateral plate mesoderm [9].

Wnt signaling is a highly complex and multiple branched signaling pathway, which plays a plethora of important roles during animal development, tissue homeostasis and in human disease [13]–[15]. During LR axis development the canonical Wnt/β-catenin pathway initiates the expression of the transcription factor *Foxj1*, a master regulator of motile cilia, in the SM [16]–[18]. The SM represents a part of the epithelial outer layer of the gastrula embryo. It neighbors the organizer caudally and involutes during gastrulation to give rise to the GRP [19]. *Foxj1* expression in Kupffer’s vesicle of zebrafish embryos is also regulated by Wnt/β-catenin [20], indicating conserved Wnt-dependency of *Foxj1* expression during LR axis development. The non-canonical Wnt/PCP pathway was shown to be necessary for cilia polarization to the posterior pole of GRP cells [21], as a prerequisite for the generation of a directed laminar flow from right to left [16], [22]. This role of Wnt/PCP for LR axis specification was also described in mouse [23], [24], arguing for evolutionary conservation of this Wnt-dependent step in LR development as well.

In zebrafish the ligands *Wnt3a*, *Wnt8* and *Wnt11* were shown to be required for LR development [20], [25], [26]. In *Xenopus*, *Wnt8a* is not expressed in the SM or GRP [27], [28]. *Wnt3a* expression only starts at stages when *Foxj1* is already expressed in the SM [29], [30]. *Wnt11b*, in contrast, is present in the oocyte and zygotic expression persists in dorsal regions before and after the onset of gastrulation [29]–[31]. *Wnt11b* can activate both canonical and non-canonical signaling branches during *Xenopus* development [32]–[34]. Maternally deposited *Wnt11b* mRNA is enriched on the dorsal side during cleavage stages and contributes to organizer formation by activation of Wnt/β-catenin signaling [31], [35]. During gastrulation and later development, *Wnt11b* and *Wnt11r* regulate convergent extension [36]–[38], neural crest cell induction and migration [39]–[41] as well as heart [42] and pronephric development [43] by activation of non-canonical Wnt signaling branches, i.e. Wnt/PCP and Wnt/calcium signaling [44], [45]. *Wnt11b* was therefore analyzed for a potential role in Wnt/β-catenin dependent *Foxj1* expression and Wnt/PCP dependent cilia polarization during *Xenopus* LR development.

## Results

### *Wnt11b* is Expressed in the Superficial Mesoderm

**Figure 1:**
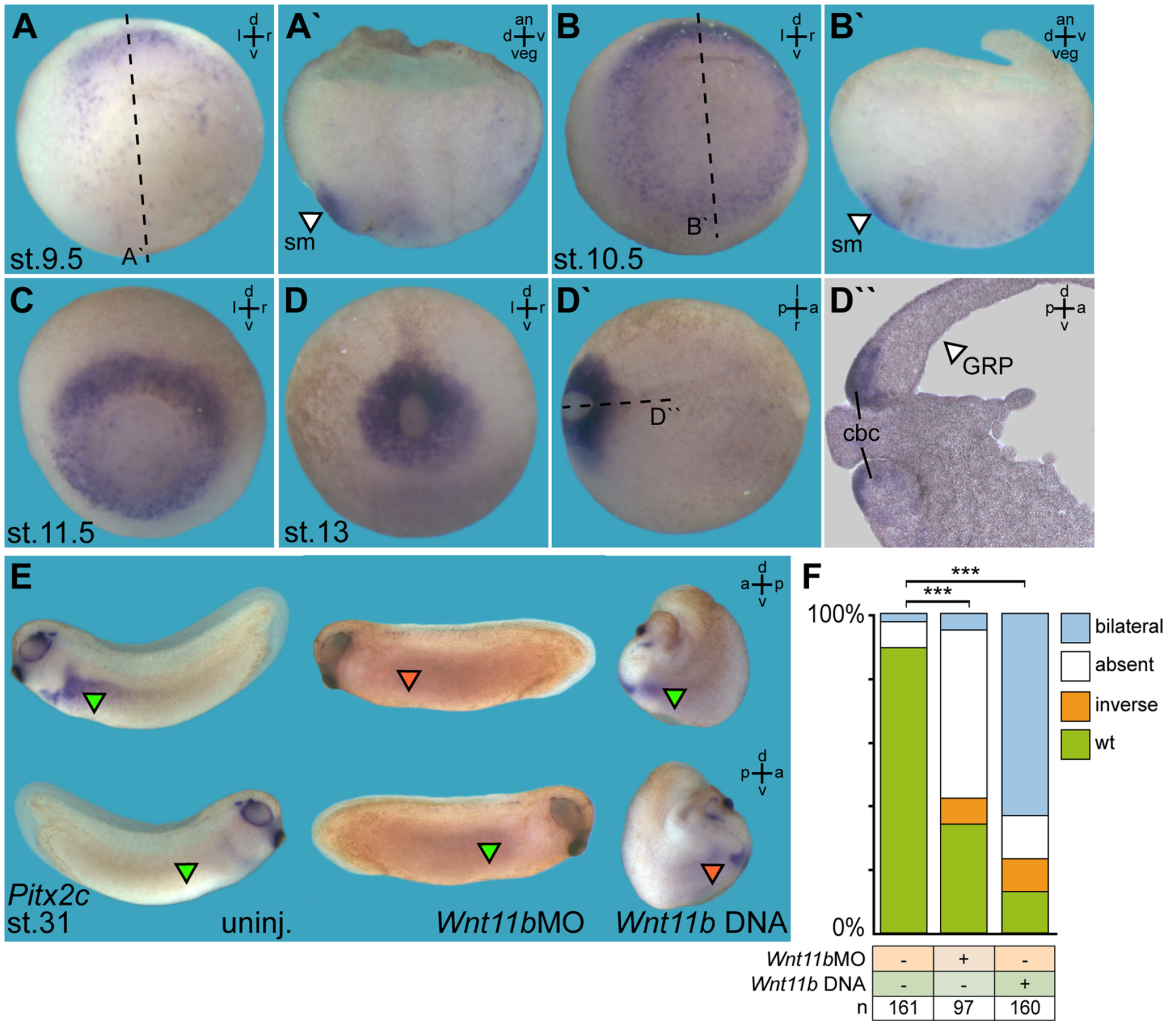
*Wnt11b* expression in the superficial mesoderm is required for asymmetric *Pitx2c* expression in the lateral plate mesoderm. (A–D) *Wnt11b* mRNA expression as determined by whole-mount in situ hybridization of staged embryos at stage (st.) 9.5 (A), st. 10.5 (B), st. 11.5 (C) and st. 13 (D). Specimens are shown in vegetal (A–D) or dorsal (D′) view. Please note that *Wnt11b* mRNA was present in the superficial mesoderm (sm; white arrowheads) on the dorsal side, as shown in bisected embryos in (A′) and (B′), while expression on the ventral side extended into deeper tissue (B′). Note also expression in the circumblastular collar (cbc) of involuting cells, as demonstrated in a histological section (D″). (E, F) Altered *Pitx2c* expression in *Wnt11b*-manipulated embryos. (E) Representative specimens. (F) Quantification of results. Green arrowheads, wild-type expression; red arrowheads, ectopic or absent expression. Dashed lines in (A, B, D′) indicate planes of section. *** Very highly significant (p<0.001). a = anterior, an = animal, d = dorsal, l = left, n = number, p = posterior, r = right, uninj. = uninjected, v = ventral, veg = vegetal.

As a first step to elucidate the role of *Wnt11b* during LR axis development we analyzed mRNA expression patterns at LR relevant sites (Figure 1A–D). With the onset of gastrulation (stage 9.5) zygotic *Wnt11b* expression started in the dorsal region of the prospective mesoderm (Figure 1A). Manual bisection of embryos revealed expression in the SM, but not in deeper layers of the organizer (Figure 1A′), reminiscent of *Foxj1* expression (Figure S1 A, A′). At stage 10.5 the domain expanded laterally (Figure 1B), eventually forming a ring of expression around the blastopore by stage 11.5 (Figure 1C). In dorsal regions the expression remained restricted to the SM, while mRNA in more lateral and ventral regions was detected in deep mesodermal cells as well (Figure 1B′). By stage 13, when the blastopore is closing, *Wnt11b* was expressed within the circumblastoporal collar (Figure 1D–D″), i.e. a ring of cells which involute into the gastrocoel. These expression patterns support a possible role of *Wnt11b* during GRP formation and LR development.

### *Wnt11b*-manipulated Embryos show Loss of Asymmetric *Pitx2c* Expression

*Wnt11b* function in LR development was addressed by morpholino oligonucleotide (MO) mediated knockdown [50], [51] of *Wnt11b* translation and in gain-of-function experiments using a full length *Wnt11b* DNA expression construct. Short of a *Xenopus* Wnt11b-specific antibody, knockdown efficiencies could not be addressed directly. SM and GRP were targeted by injecting 4-cell embryos into the dorsal marginal zone [52]. Specimens were cultured until they reached stage 31 and analyzed for *Pitx2c* gene expression by whole mount in situ hybridization [2], [53]–[55]. Remarkably, both gain and loss of *Wnt11b* function resulted predominantly in loss of asymmetric *Pitx2c* expression in the left LPM (Figure 1E, F). In *Wnt11b* morphants *Pitx2c* expression was mostly absent, while bilateral expression of *Pitx2c* represented the most frequently encountered phenotype following *Wnt11b* DNA injection (Figure 1E, F). Ectopic expression of *Wnt11b* in addition resulted in severely shortened anterior-posterior axes (Figure 1E), indicative of convergent extension defects [56].

Next we asked whether changes in *Foxj1* expression might correlate with absence of *Pitx2c* expression in morphants. Unexpectedly, differences from the wildtype pattern were recorded only in a minority of cases (Figure S1B, C). As both organizer function and notochord formation are required for normal LR development, we analyzed *Xnr3* and *Not* mRNA expression in *Wnt11b* morphants [18], [57], [58]. Both were expressed in wildtype fashion (Figure S1D, E), demonstrating that organizer and notochord were not affected.

In order to test whether ligand-mediated Wnt signaling was indeed required for *Foxj1* induction in the SM, we used a previously characterized antisense MO to interfere with translation of the canonical Wnt receptor *Frizzled 8* (*Fz8*), which was shown to be active on the dorsal side of the *Xenopus* gastrula [59], [60]. As shown in Figure S1 (F, G), *Foxj1* expression in the SM was severely affected in *Fz8* morphants, and down-regulation was rescued by co-injection of a β-catenin DNA expression construct. These experiments confirm our previous results that ligand-mediated canonical Wnt signaling is required for *Foxj1* expression in the SM [16]. *Wnt11b* thus is required in a *Foxj1* independent manner in Wnt-dependent LR axis development.

### Manipulation of *Wnt11b* Perturbs Leftward Flow at the GRP

**Figure 2:**
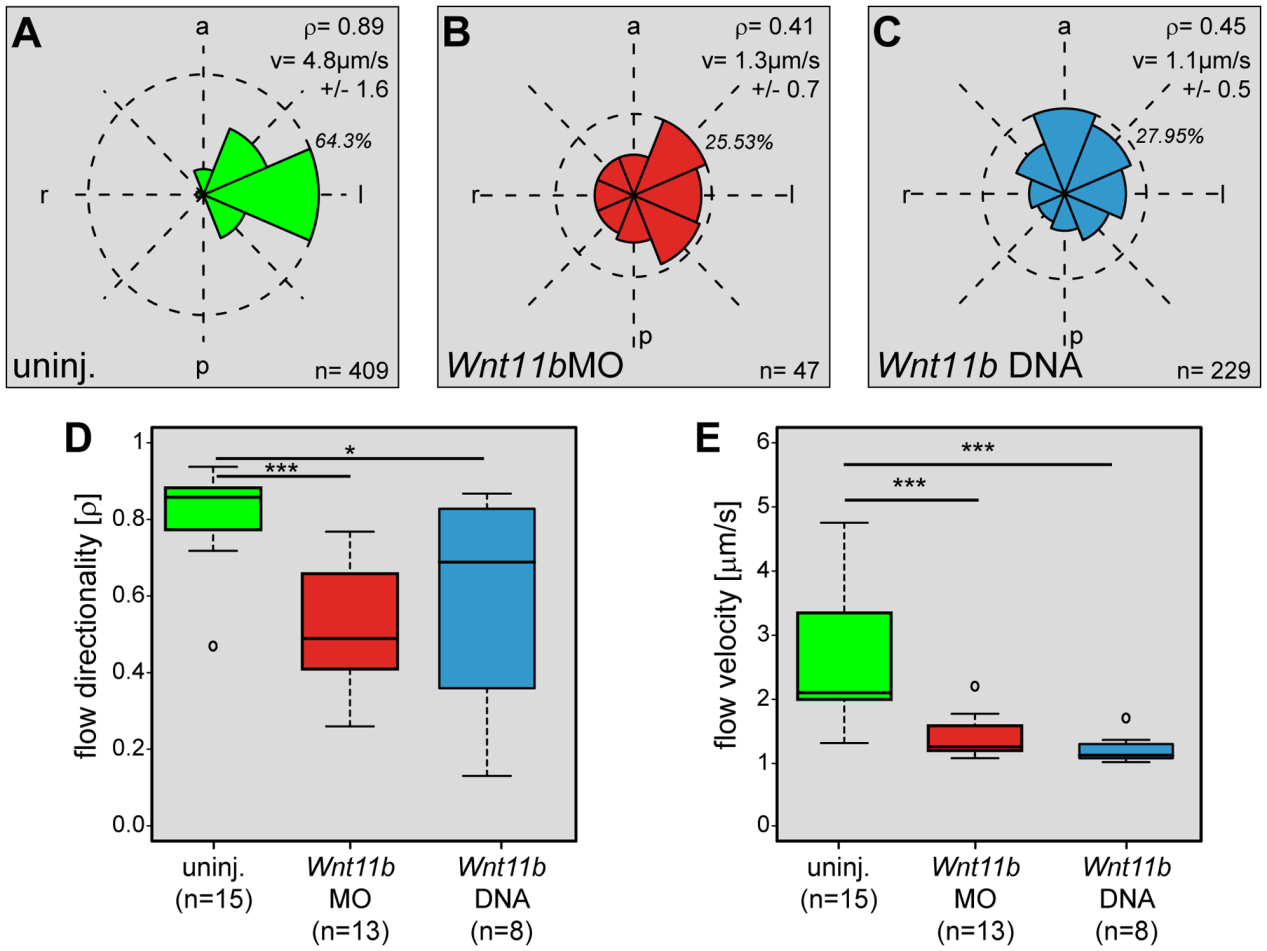
*Wnt11b* is required for leftward flow at the GRP. Flow analysis in wildtype and manipulated specimens. (A–C) Frequency distribution of trajectory angles in representative explants of uninjected control embryos (uninj., A), *Wnt11bMO* (B) and *Wnt11b* DNA (C) injected specimens. Dashed circles indicate maximum frequency (in %), n represents the number of tracked particles above threshold. a = anterior, l = left, p = posterior, r = right, v = average velocity of particles, ρ = quality of flow. (D, E) Compiled results of all embryos analyzed for flow directionality (D) and velocity of fluorescent beads added to GRP explants at stage 17 (E). Note that both parameters were significantly reduced in *Wnt11b* morphants or *Wnt11b* DNA injected embryos. In (D, E), n represents number of analyzed explants. * Significant (p<0.05), ***Very highly significant (p<0.001), n = number of analyzed explants.

In an attempt to systematically dissect the LR pathway in *Wnt11b* manipulated embryos, we turned to leftward flow as the next step downstream from SM specification and *Foxj1* induction. Directionality and velocity of flow were analyzed in dorsal explants of *Wnt11bMO* and *Wnt11b* DNA injected specimens (Figure 2 and Movie S1). Robust leftward flow was detected in uninjected control explants (Figure 2A), while flow directionality was compromised in *Wnt11b* morphants (Figure 2B) as well as following *Wnt11b* DNA injection (Figure 2C). To evaluate flow in groups of manipulated specimens we used the dimensionless number rho (ρ), which provides a qualitative measure (Figure 2D). Rho was calculated from time-lapse movies and represents the mean resultant directionality of particle trails (Rayleigh’s test of uniformity) [5]. Rho values range from 1, when all trajectories point in the same direction, to 0, when particles move randomly. Explants from uninjected control embryos showed a mean ρ-value of 0.82±0.12, while flow in *Wnt11bMO* and *Wnt11b* DNA injected specimens reached ρ-value of 0.51±0.17 and 0.6±0.28, respectively. While these data clearly demonstrate the impact of *Wnt11b* knockdown on flow directionality, the residual flow has not lost directionality altogether. Flow velocities were calculated from the same set of time-lapse movies (Figure 2E). Velocities in *Wnt11b* morphants and following *Wnt11b* DNA injection were found at 1.37µm/s±0.32 and 1.19µm/s±0.23, respectively, compared to uninjected controls which displayed a mean velocity of 2.54µm/s±0.94 (Figure 2E). Velocity thus was affected in a more pronounced manner than flow directionalty. These data pinpoint flow as a decisive step for *Wnt11b* function during LR development.

### *Wnt11b* Regulates Wnt/PCP Dependent Cilia Polarization and Morphogenesis of the GRP

**Figure 3:**
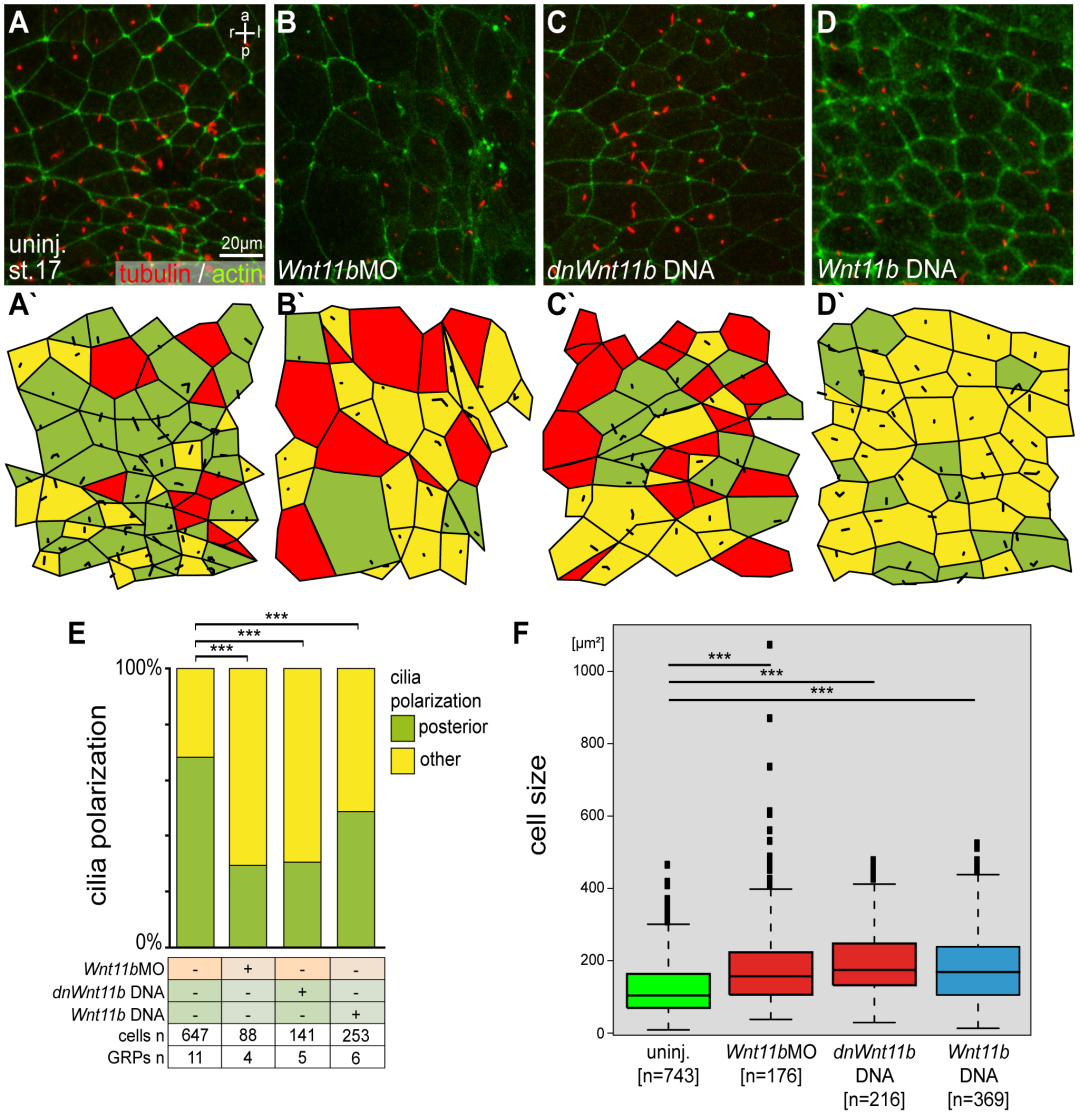
*Wnt11b* is required for cilia polarization and GRP morphogenesis. Embryos were injected at the 4-cell stage into the prospective dorsal marginal zone and dorsal explants were prepared at stage 17. Specimens were processed for immunohistochemistry (IHC) to assess cilia polarization, ciliation rate and cell surface area. (A–D) Presence and polarization of cilia, as shown by acetylated tubulin IHC to stain cilia (red) and phalloidin to stain actin (green) in order to outline cell boundaries. (A) Control uninjected (uninj.) specimen. (B) *Wnt11b* morphant. (C) Specimen injected with dominant-negative *Wnt11b* DNA (*dnWnt11b*). (D) Specimen injected with wild-type *Wnt11b* DNA. (A′–D′) Evaluation of ciliation and polarization. Green = posterior localization of cilia, yellow = other localization, red = cells without cilia. (E, F) Evaluation of results. (E) Cilia polarization. (F) Apical cell surface area. ***Very highly significant (p<0.001). a = anterior, l = left, p = posterior, r = right.

Next we analyzed cilia polarization, a process known to depend on Wnt/PCP [21]–[24]. GRP explants from *Wnt11b* manipulated embryos were stained for cilia and cell borders using an antibody against acetylated tubulin and phalloidin (Figure 3A–D). In uninjected control GRPs most cells were ciliated (79%) and cilia were localized to the posterior pole (Figure 3A′, E). Analysis of *Wnt11b* morphants and embryos injected with DNA encoding either a dominant negative *Wnt11b* construct (*dnWnt11b*; [37]), which is specific for Wnt5/11-type ligands without affecting the canonical pathway, or wildtype *Wnt11b* revealed disturbed cilia polarization (Figure 3B′–D′ and E). Remarkably, clear differences were seen between loss-of-function scenarios and ectopic expression of *Wnt11b*. The ciliation rate was reduced to 50% and 66% in *Wnt11b* morphants and following injection of *dnWnt11b* DNA, respectively. Overexpression of Wnt11b did not alter the wildtype ciliation rate of about 80%, but cilia were predominantly unpolarized, i.e. arose in a central position (Figure 3D′, E). In addition we observed that the apical surface of GRP cells was enlarged upon *Wnt11b* manipulation (Figure 3F). Average surface areas measured 193.71µm^2^±143.00, 195.79µm^2^±92.33 and 178.64µm^2^±95.73 in *Wnt11bMO*, *dnWnt11b* DNA and *Wnt11b* DNA injected specimens, respectively, compared to 123.88µm^2^±74.28 in control specimens, indicating an effect on GRP cell morphogenesis (Figure 3F). Taken together, balanced levels of *Wnt11b* seem to be required for Wnt/PCP dependent cilia polarization and GRP morphogenesis.

### Loss of *Wnt11b* Disrupts *Xnr1* and *Coco* Expression in Lateral Sensory GRP Cells

**Figure 4:**
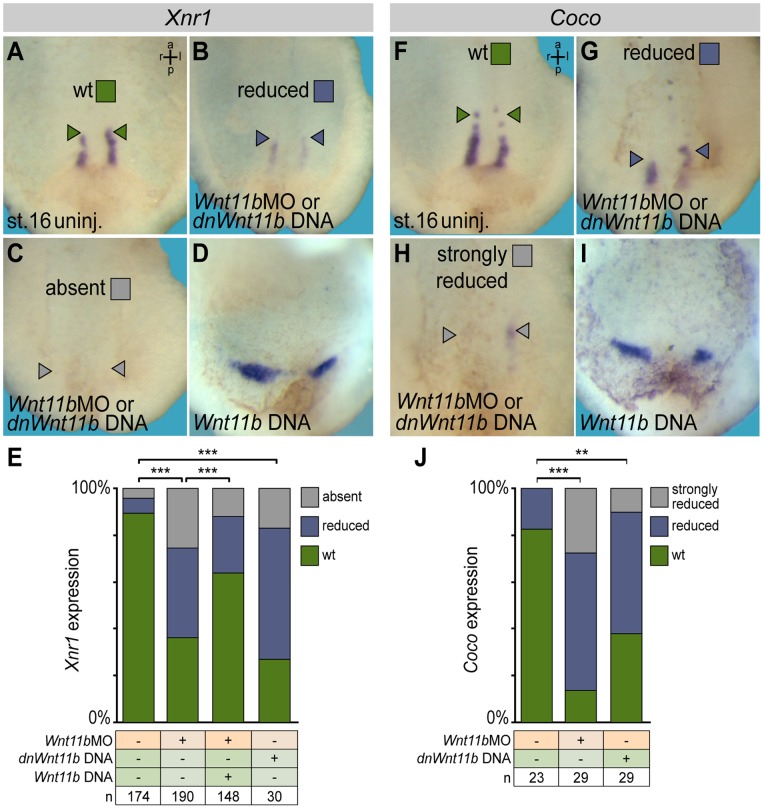
Altered *Xnr1* and *Coco* expression in *Wnt11b* manipulated embryos. Embryos were injected at the 4-cell stage into the DMZ and analyzed for *Xnr1* (A–E) or *Coco* expression (F–J) by whole mount in situ hybridization. Wildtype expression patterns (A, F) were reduced (B, G) or absent/strongly reduced (C, H) in *Wnt11b* morphants and in embryos injected with a *dnWnt11b* DNA construct, while signal intensities were unaltered upon ectopic wildtype *Wnt11b* expression from a DNA construct (D, I). (E, J) Quantification of results. Note that co-injection of wild-type *Wnt11b* DNA was sufficient to partially rescue *Xnr1* expression at the GRP of *Wnt11b* morphants. ***Very highly significant (p<0.001). a = anterior, l = left, p = posterior, r = right.

GRP analyses implemented *Wnt11b* in the LR cascade at the level of flow or events downstream. They do, however, not provide an explanation as to the opposing effects of *Wnt11b* manipulation on *Pitx2c* expression, namely absence in morphants and bilateral induction upon ectopic expression (cf. Figure 1E, F). Our previous analysis of *ATP4a* has shown that a turbulent and attenuated cilia-driven flow is sufficient to induce the nodal-cascade in a bilateral fashion [16], in line with the characterization of *Wnt11b* DNA injected specimens presented here (Figure 1E, F and 2C). In order to elucidate the opposing effect in morphants, we analyzed the lateral GRP cells which express both *Xnr1* and its inhibitor *Coco* (Figure 4A, F), and which are required for LPM *Xnr1* induction [9]. *Wnt11b* morphants and specimens injected with dnWnt11b showed significantly reduced expression levels of both genes (Figure 4B, C, G, H). Ectopic expression of *Wnt11b*, in contrast, showed comparable signal strength to wildtype specimens (Figure 4D, I), although domains were not aligned in parallel due to more pronounced convergent-extension phenotypes encountered in these experiments (cf. Figure 1E). Specificity of treatments was confirmed by co-injection of *Wnt11b* DNA in *Wnt11b* morphants, which partially restored *Xnr1* expression (Figure 4E). These differential effects on *Xnr1*/*Coco* provide an explanation for LPM *Pitx2c* induction in the various experiments. Taken together, our data involve *Wnt11b* in the setup of the GRP and leftward flow.

## Discussion

A role of Wnt signaling in LR axis development has been previously demonstrated in vertebrate model organisms including *Xenopus* [16], [20]–[26], [49], [61]–[70]. The sequential activity of two Wnt pathway branches is required for cilia-driven leftward flow: (1) canonical Wnt/β-cat signaling regulates *Foxj1* expression during gastrulation in the *Xenopus* SM and in zebrafish Kupffer’s vesicle [16], [20]; (2) non-canonical Wnt/PCP signaling is required for the posterior alignment of motile cilia at the frog GRP and the posterior notochord in mouse [16], [21], [23], [24]. The present work confirmed the *Fz8*-mediated Wnt/β-catenin dependent activation of *Foxj1* expression in the SM [16]. *Wnt11b*, however, contributes only marginally, if at all, to this process. Two additional canonical Wnt ligands are expressed during *Xenopus* gastrulation, *Wnt3a* and *Wnt8a* [27]–[30]. *Wnt3a* expression starts only after the onset of *Foxj1* transcription in the SM. It is therefore tempting to speculate that Wnt8a represents the main canonical activator of *Foxj1* expression during gastrulation. This notion may seem contra-intuitive at first glance, as *Wnt8a* is expressed in ventral and lateral portions of the prospective mesoderm but not in the SM itself [27]–[28]. We have, however, previously shown that ventral and lateral portions of the mesodermal ring are competent to express *Foxj1* upon activation of canonical Wnt signaling [16]. Restriction of *Foxj1* expression to the SM thus might be mediated by expression of the receptor *Fz8*.

Our study clearly demonstrates a role of *Wnt11b* in Wnt/PCP dependent cilia polarization at the GRP. Cilia alignment was altered by both gain and loss of non-canonical *Wnt11b* signaling, in agreement with findings in other systems in which non-canonical Wnt signaling was manipulated [16], [21], [23], [24]. Although the role of Wnt/PCP in *Vangl2*-dependent cilia polarization is well established [21]–[24], the initial global cue(s) for posterior orientation of cilia in vertebrate flow-generating epithelia has not been identified as yet. *Wnt11b* is expressed in the circumblastoporal collar, i.e. in cells en route to involute into the gastrocoel. Cells expressing *Wnt11b* mRNA therefore likely start secreting the protein following involution, i.e. when they are localized at the posterior margin of the GRP. Such a localization might establish a posterior to anterior gradient of Wnt11b protein, which, together with the co-expressed non-canonical ligand Wnt5a [32], [37], [38] might serve as instructive cue for cilia polarization at the GRP. Wnt gradients might mediate asymmetric phosphorylation of Vangl2, leading to anterior-posterior asymmetric localization of motile cilia, similar to the polarization mechanism proposed in the mouse limb bud [71].

Remarkably, we found another Wnt-dependent process during LR axis formation in *Xenopus*, namely *Xnr1*/*Coco* expression at the lateral-most aspects of the GRP. Previous reports have implicated canonical Wnt/β-cat signaling in the expression of the *Xnr1*/*Coco* homologs *spaw*/*charon* in zebrafish [25], [62] and in the homologous *nodal* expression domain in mouse [65]. Canonical Wnt signaling is important for organizer formation and function, which in turn is required for correct LR axis development [57]. The observed effects on nodal expression therefore might be indirect. The unaltered *Xnr3* and *Not* expression patterns in our *Wnt11b* morphants and the effectiveness of a *dnWnt11b* DNA construct, which is only activated post MBT [72], however, argue for a specific impact of Wnt signaling on lateral cells of the GRP, unrelated to organizer function and notochord formation.

We have recently shown that *ATP4a* is required for Wnt/β-catenin and Wnt/PCP signaling during *Xenopus* LR development [16], similar to *ATP6* [73]–[75]. It seems unlikely that *Wnt11b* acts on *Xnr1*/*Coco* expression via the Wnt/β-catenin or Wnt/PCP pathways, because morpholino-mediated loss of *ATP4a* function did not affect *Xnr1* or *Coco* expression. Furthermore, pharmacological inhibition of *ATP6* during frog, chick and zebrafish development did not lead to a loss, but randomized *Xnr1*, *nodal* or *spaw* expression, respectively [76].

Which signaling branch might Wnt11b act on in the context of LR development? We like to propose an involvement of Wnt/calcium signaling. *Wnt11b* is known to interact with the Wnt/calcium pathway during *Xenopus* development, especially during gastrulation [43], [45], [77]. Manipulation of calcium signaling during gastrulation alters LR development in zebrafish, *Xenopus* and mouse [63], [78]–[81], in line with a possible role of Wnt/calcium signaling in the regulation of *Xnr1* and *Coco* expression. Further experiments are required in the various model organisms to resolve the precise mechanism of Wnt-dependent expression of *nodal* and its respective inhibitor in the lateral flow-sensing cells of the ciliated organs of laterality.

## Materials and Methods

### Ethics Statement

All animals were treated according to the German regulations and laws for care and handling of research animals, and experimental manipulations according to §6, article 1, sentence 2, nr. 4 of the animal protection act were approved by the Regional Government Stuttgart, Germany (Vorhaben A 365/10 ZO “Molekulare Embryologie”).

### Statistical Evaluation of Results

Statistical evaluation of experiments represented by bar graphs was performed using chi-square tests (http://www.physics.csbsju.edu/stats/contingency.html). In Figure 1F, the number of manipulated embryos (*Wnt11b*MO or *Wnt11b* DNA) displaying wt, inverse, bilateral or absent *Pitx2c* expression was compared to the numbers of embryos displaying these expression patterns in uninjected control specimens. In Figure S1C, F, the number of morphant embryos with wt, reduced and absent *Foxj1* expression was compared to the number of embryos with wt, reduced or absent expression in uninjected controls and embryos co-injected with β-catenin DNA. In Figure 4E, J, the number of *Wnt11b*MO or *dnWnt11b* DNA injected embryos with wt, reduced or absent/strongly reduced expression of *Xnr1* or *Coco*, respectively, was compared to the number of embryos with these characteristics in uninjected control specimens and *Wnt11b* morphants co-injected with wt *Wnt11b* DNA. Statistics of experiments represented by box plots were calculated by Wilcoxon sum of ranks (Mann-Whitney) tests (http://www.fon.hum.uva.nl/Service/Statistics/Wilcoxon_Test.html).

### Manipulation of Embryos

Embryos were injected at the two- to four-cell stage using a Harvard Apparatus setup in 1× modified Barth’s solution (MBSH) with 4% Ficoll (BioChemica) and transferred to 0.1× MBSH 15 min after injection. Drop size was calibrated to about 7–8 nl per injection. Rhodamine-B or Cascade blue dextran (0.5–1.0 mg/ml; Molecular Probes) were coinjected and used as lineage tracer. *Wnt11bMO* (5′-TAACCCAGTGACGGGTCGGAGCCAT-3′) was used at 1–2 pmol per embryo, *Fz8MO* [46] at 2 pmol per specimen. DNAs were purified using the PureYield Plasmid Midiprep kit (Promega) and diluted to a concentration of 1 ng/µl (*dnWnt11b* CS2+ [38]), 0.5–1 ng/µl (*Wnt11b* CS2+ [38]) and 1ng/µl (*β-cat:GFP* [47]).

### Whole-Mount In Situ Hybridization

Embryos were fixed in MEMFA for 1–2 hrs and processed following standard protocols. Digoxigenin-labeled (Roche) RNA probes were prepared from linearized plasmids using SP6, T3, or T7 RNA polymerase (Promega). In situ hybridization was conducted as previously described [16]. For histological analyses, embryos were embedded in gelatin-albumin and sectioned on a vibratome (30 µm).

### Flow Analysis

Embryos were coinjected with lineage tracer to control for correct targeting of the GRP. Data processing was as previously described [5], [16], [48], [49]. The whiskers of the box plots extend to maximal 1.5×IQR, and outliers are displayed as circles.

### Immunohistochemistry and GRP Analysis

Immunohistochemistry was performed as described [48] using Anti-Tubulin Acetylated (mouse, 1∶700; Sigma) and anti-mouse Cy3 (sheep, 1∶250; Sigma). Cell boundaries were visualized by Alexa 488-conjugated phalloidin (Invitrogen), which stained the actin cytoskeleton. Imaging was performed on a Zeiss LSM700. To determine GRP cell parameters, an area of 320×320 µm at the center of the GRP was selected for manual analysis of cilia number/polarization and GRP cell size using ImageJ [16], [49]. The whiskers of the box plots extend to maximal 1.5× IQR, outliers are displayed as circles.

## Supporting Information

Figure S1 *Foxj1* expression requires Wnt signaling through *Fz8*, but is largely independent of *Wnt11b*. (A, A′) *Foxj1* expression in the superficial mesoderm at stage (st.) 10.5. in whole mount (A) and bisected specimens (A′). (B, C) Marginal effects on *Foxj1* mRNA expression levels and localization in *Wnt11b* morphants (quantification in C). (D, E) Wildtype expression of *Xnr3* (D) and *Not* (E) in *Wnt11b* morphant embryos. (F, G) *Foxj1* expression requires *Fz8*. (F) Summary of results. (G) Altered *Foxj1* expression in *Fz8* morphants is partially rescued by co-injection of *β-catenin* (*β-cat*). Green arrowhead, wild-type expression; red arrowhead, reduced expression; gray arrowhead, absent expression. Dashed line in (A) indicates plane of bisection. ** Highly significant (p<0.01), *** Very highly significant (p<0.001). a = anterior, an = animal, d = dorsal, l = left, n = number, p = posterior, r = right, v = ventral, veg = vegetal.
(TIF)

Movie S1 Flow defects in GRP explants from *Wnt11b* manipulated embryos. Movie shows time-lapse sequences of dorsal explants to which fluorescent beads were added (cf. Figure 2A-C). Specimens were mounted dorsal side down and viewed from the ventral side, anterior to the top. Movie represents a total length of 500 frames taken at a rate of 2 frames/sec and runs at 40×real time. Opening frame displays bright field images and indicates orientation of GRP (dashed lines). Videos were processed to yield gradient time trails (GTTs), i.e. color-coded tracks of beads which revealed direction of transport and velocity of particles (from green to red; 25 s). Note that robust leftward flow (uninjected controls) was impaired in *Wnt11b* morphant and upon injection of wild-type *Wnt11b* DNA.
(MOV)
