# Supplementary figures and images for: Wnt11b Is Involved in Cilia-Mediated Symmetry Breakage during Xenopus Left-Right Development
Source: PLoS One. 2013 Sep 13;8(9):e73646. doi: 10.1371/journal.pone.0073646 (PMC3772795; doi:10.1371/journal.pone.0073646)

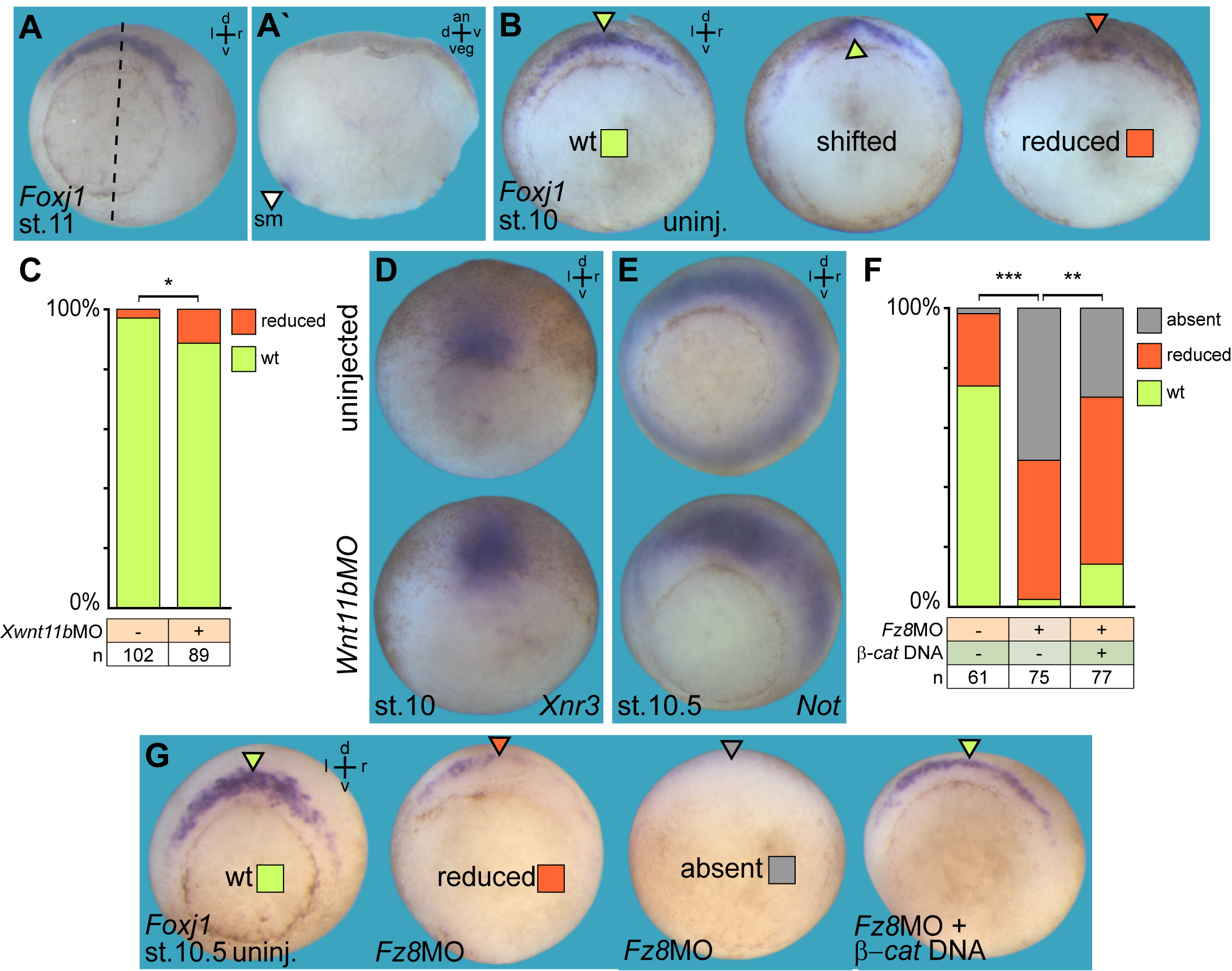

Supplement: Figure S1 — Foxj1 expression requires Wnt signaling through Fz8, but is largely independent of Wnt11b. (A, A′) Foxj1 expression in the superficial mesoderm at stage (st.) 10.5. in whole mount (A) and bisected specimens (A′). (B, C) Marginal effects on Foxj1 mRNA expression levels and localization in Wnt11b morphants (quantification in C). (D, E) Wildtype expression of Xnr3 (D) and Not (E) in Wnt11b morphant embryos. (F, G) Foxj1 expression requires Fz8. (F) Summary of results. (G) Altered Foxj1 expression in Fz8 morphants is partially rescued by co-injection of β-catenin (β-cat). Green arrowhead, wild-type expression; red arrowhead, reduced expression; gray arrowhead, absent expression. Dashed line in (A) indicates plane of bisection. ** Highly significant (p<0.01), *** Very highly significant (p<0.001). a = anterior, an = animal, d = dorsal, l = left, n = number, p = posterior, r = right, v = ventral, veg = vegetal. (TIF) [file pone.0073646.s001.tif]
